# The role of individual and regional environment factors on levels of a cardiovascular risk predictor in middle-aged and older Chinese adults

**DOI:** 10.7189/jogh.15.04131

**Published:** 2025-05-16

**Authors:** Kexin Li, Peihan Wang, Zhenbo Wang, Chengdong Xu, Shaobin Wang, Zhiyi Li, Peng Wang

**Affiliations:** 1 Key Laboratory of Land Surface Pattern and Simulation, Institute of Geographic Sciences and Natural Resources Research, Chinese Academy of Sciences, Beijing, PR China; 2 Key Laboratory of Regional Sustainable Development Modelling, Institute of Geographic Sciences and Natural Resources Research, Chinese Academy of Sciences, Beijing, PR China; 3 University of Chinese Academy of Sciences, Beijing, PR China; 4 State Key Laboratory of Resources and Environmental Information System, Institute of Geographic Sciences and Natural Resources Research, Chinese Academy of Sciences, Beijing, PR China

## Abstract

**Background:**

Cardiovascular disease (CVD) remains the leading cause of death in China and worldwide. However, a large proportion of CVD can be prevented by regulating the levels of cardiovascular risk predictors. Despite the contribution of well-established factors to changes in cardiovascular risk predictors, the role of the regional environment and its combined effects with individual factors, which could affect health outcomes, remain unclear.

**Methods:**

We included 10 308 middle-aged and older Chinese adults from the 2015 China Health and Retirement Longitudinal Study. High-sensitivity C-reactive protein (hs-CRP) is a cardiovascular risk predictor. Related potential factors including individual characteristics, regional air pollution, and regional socioeconomic status characteristics were also collected. The geographical detector method was used to quantify the explanatory power of individual and regional factors separately and in pairs in the hs-CRP levels according to regions (southern *vs*. northern China).

**Results:**

Blood triglyceride had the highest explanatory power for hs-CRP levels. Regional environment factors, including air pollution and socioeconomic status, significantly affected hs-CRP levels, and the results differed by region. Indoor air pollution and regional industrial structure had a stronger effect on hs-CRP levels in the south, whereas outdoor air pollution and economic level had a greater effect in the north. The interactions between any two of the paired factors enhanced the effects.

**Conclusions:**

Spatial stratified heterogeneity of the leading risk factors for hs-CRP, a powerful cardiovascular risk predictor, was found. The combined effect of individual factors and regional environment enhanced the explanatory power of each risk factor. The results suggest that policymakers should choose different optimal approaches to regulate the cardiovascular risk predictor levels of middle-aged and older Chinese adults in different regions and the interaction effects between individual factors and the regional environment should be considered.

Cardiovascular disease (CVD) is the leading cause of morbidity and mortality in China and worldwide, posing a significant public health challenge, particularly for economically disadvantaged populations [1,2]. Most CVD cases can be prevented by addressing cardiovascular risk predictors [3,4]. High-sensitivity C-reactive protein (hs-CRP) is an independent predictor of cardiovascular risk [5,6]. Mildly elevated hs-CRP is associated with increased CVD risk in apparently healthy people [7,8]. While attempts have been made to reduce hs-CRP levels to prevent CVD [4,9,10], effective clinical approaches to directly lower hs-CRP have only recently emerged [4]. It is important to address risk factors associated with elevated hs-CRP levels to maintain healthy levels. The risk factors of elevated hs-CRP have been extensively studied, focusing predominantly on air pollution [11–17]. Contrarily, the role of regional environment, such as socioeconomic status (SES), has rarely been evaluated, and little is known about the combined effect of individual factors and regional SES. Studies suggest that overlooking regional differences and their interactions with individual factors can underestimate or overestimate their health impacts [18,19].

High-sensitivity C-reactive protein levels are associated with age, gender, obesity, blood lipid levels (triglyceride (TG) and high-density lipoprotein (HDL)), smoking, depression [11,12], alcohol use [11,20], and air pollution (particulate matter (PM_2.5_, PM_10_) and ozone) [13,15–17]. Studies show a strong link between PM_2.5_ pollution and hs-CRP levels [17] but findings on environmental impacts have been inconsistent [15,17,21–24] due to regional heterogeneity.

The regional imbalance in SES, such as gross domestic product (GDP) *per capita*, industry structure, social capacity of healthcare, plays a key role in health disparities and can manifest directly or through interactions with other risk factors (*e.g*. lipid levels and PM_2.5_) [25–29]. A study explored the existence of regional socioeconomic differences in the association between long-term exposure to PM_2.5_ and mortality [18]. Another study demonstrated that regional socioeconomic deprivation affected the association between individual-level risk factors and hs-CRP concentrations in adults aged 18–79 years in Germany [19]. In China, rapid development and regional heterogeneity show how SES might affect hs-CRP concentrations and, consequently, CVD risk; however, these aspects remain underexplored. China’s rapidly aging population [1,30] highlights the urgency to identify the leading risk factors influencing hs-CRP across different regions. Such insights may guide policymakers to adopt efficient and cost-effective ways to regulate hs-CRP levels and control CVD, reducing loss of economy in society. However, the leading risk factors of hs-CRP and its regional variability in China are underexplored.

Most existing studies employed correlation or regression analysis to explore the factors influencing hs-CRP levels. As such, in subsequent quantitative analysis of the exposure-response relationship, confounding factors were included or excluded [19,31,32]. Although, these methods can characterise the effect of factors on hs-CRP levels, they are often more suitable for measuring the linear part of the association and cannot simultaneously measure the nonlinear part. Meanwhile, the regression methods can only express individual correlation between the dependent and explanatory variables, but cannot describe their interactions. Also, correlation and regression analyses fail to reveal the spatial heterogeneity of factor effects. As hs-CRP levels are influenced by multiple factors, the interactions between various factors and spatial differentiation of factor effects, need to be considered. Geographical detector, a mature spatial statistical model, has been used to reach the target [33–35]. Based on spatial stratification heterogeneity, the geographical detector assumes that if X (explanatory variables) and Y (dependent variable) have spatial correlation, their spatial distribution are consistent, and X will have a determinant power (q-statistic) on Y. If the relationships between X and Y are inconsistent within different strata, then the factors (X) that form these strata are confounding factors, and could be controlled by stratification. Thus, geographical detector is unaffected by variable collinearity or potential confounding variables not included. Each selected factor is independently input for the interpretation of the dependent variable and their contributions were investigated by testing spatial variance [34,35]. This method can further explore the interaction between two explanatory variables with respect to the dependent variable, and it has been applied in many fields, such as human health [33–38].

We aimed to explore the spatial variation of the effect of possible risk factors for hs-CRP levels in middle-aged and older Chinese adults using a nationally representative investigation. We investigated the effect of GDP, healthcare, and other regional SES factors on hs-CRP levels in middle-aged and older adults and explored their interaction with individual factors (*e.g*. lipid levels), and air pollution. Our findings offer insight into developing optimal approaches for regulating hs-CRP levels and CVD preventing.

## METHODS

### Data sources

We used data from the 2015 wave of the China Health and Retirement Longitudinal Study (CHARLS), an ongoing and nationally representative survey of Chinese adults aged ≥ 45 years designed to examine aging-related issues in China [39]. It was conducted by the National School for Development (China Centre for Economic Research) at Peking University and approved by the Biomedical Ethics Review Committee of Peking University (the IRB approval number for the main household survey, including anthropometrics, is IRB00001052-11015; the IRB approval number for biomarker collection was IRB00001052-11014). All participants provided their informed consent.

### Study design and study population

Approximately 21 095 individuals participated in the 2015 wave. We excluded the following participants:

1) 7744 without hs-CRP, triglyceride, and HDL data

2) 512 with hs-CRP levels > 10 milligrammes per litre (mg/L), values above which could be due to acute infections or other chronic inflammatory conditions [40]

3) 206 with missing body mass index (BMI) data [41]

4) 14 with missing data on smoking habits

5) 14 with missing data on drinking habits

6) 2297 in Ganzi, Liangshan, Chuxiong, Chaohu, Qiannan, Qiandongnan, Xilingol, Aksu, Hinggan, Jiangmen, Yuncheng, and Enshi with lacking SES and air pollution data.

**Figure 1:**
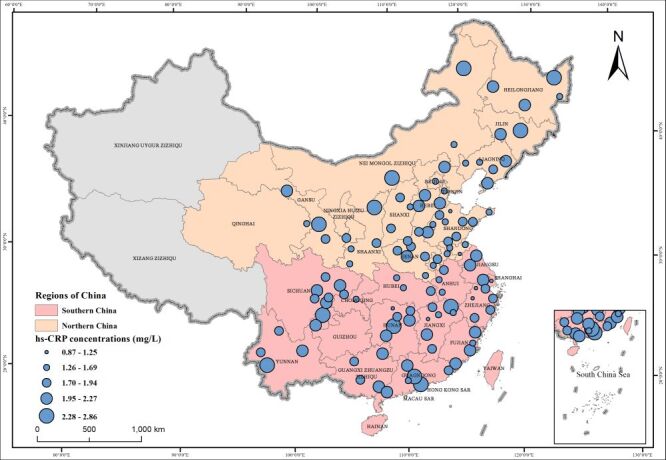
Locations of the study sites with the average hs-CRP concentrations in middle-aged and older Chinese adults (2015). hs-CRP – High-sensitivity C-reactive protein.

Overall, 10 308 participants who completed both questionnaires and blood tests from 111 cities in 26 provinces in China were included (**Figure 1**). We divided China into north and south according to the traditional geographical division, with the Qinling Mountains to the west and the Huai River to the east [42,43] (Table S1 in the **Online Supplementary Document**).

The circle size represents the average hs-CRP concentrations in middle-aged and older Chinese adults from each study site, with larger circles indicating higher concentrations (**Figure 1**). Xinjiang Uygur Zizhiqu and Xizang Zizhiqu are excluded here.

The selected potential risk factors of hs-CRP levels included individual factors, air pollution, and regional SES factors. Figure S1 in the **Online Supplementary Document** shows the relationship between hs-CRP concentrations and their proxy variables.

### Measurement

High-sensitivity C-reactive protein and blood-related factors: Venous blood samples were collected by the Center for Disease Control (CDC) station during the investigation. The Venous blood samples were divided into plasma and grey plasma after treatment. The plasma samples were transferred into three 0.5 mL cryogenic vials, immediately cryopreserved at −20°C and transported to the Chinese Centre for Disease Control and Prevention in Beijing within two weeks. They were placed in a deep refrigerator in the laboratory of Capital Medical University and stored at −80°C. High-density lipoprotein and TG was detected by enzymatic colorimetry at the Youanmen Centre for Clinical Laboratory at Capital Medical University, while hs-CRP was detected by immunoturbidimetry [39].

#### Individual factors

Demographic characteristics were collected using the CHARLS questionnaire for wave 2015. Health status, such as obesity and depressive symptoms, and health behaviours, such as drinking and smoking, were also collected, all of which have been linked to hs-CRP levels [44]. Obesity was determined using BMI [41]. Depressive symptoms were evaluated using the Center for Epidemiological Studies Depression 10 Scale [45]. Regarding smoking and alcohol habits, participants were classified as never, former, and current smokers or drinkers.

#### Air pollution

Outdoor air pollution was the annual average concentrations of PM_2.5 _in 2015 at the participants’ residential addresses (city level). The PM_2.5_ dataset in 2015 for all cities included in this study was from environmental observation data from the stations of the China National Environmental Monitoring Centre. The PM_2.5_ levels are published hourly on the China National Environmental Monitoring Centre website. The daily average values in each city were aggregated, and the annual average values were calculated from the daily averages.

Indoor air pollution was determined using the types of fuel used for cooking by the respondents [46,47], which were collected from the CHARLS questionnaire for wave 2015. The fuel types in CHARLS were categorised as clean fuels (liquefied gas, natural gas, and electricity), solid fuels (coal, biomass charcoal, wood, and straw), and others.

#### Regional SES factors

Gross domestic product *per capita* is one of the most important SES factors for improving life expectancy and health condition [48]. Thus, cities’ GDP *per capita* was selected to represent the macro-level economic level and was obtained from the National Bureaus of Statistics of China 2015. The cities’ proportion of tertiary industries was selected as an indicator of industrial structure. Industrial structure can indirectly affect the health of residents by affecting the regional ecological environment and economic development [29,49]. With reference to healthcare capacity, the number of doctors per 10 000 individuals was included. Healthcare capacity affects the health of residents, especially the doctor density [50,51]. The proportion of tertiary industry and the number of doctors per 10 000 individuals were derived from the China City Statistical Yearbook [52].

### Statistical analysis

We used Student’s t-test to compare the hs-CRP levels between regions and the subgroups divided by age and gender. The impact of selected risk factors (Figure S1 **in the Online Supplementary Document**) and their interactions were assessed using the geographical detector method.

The q-statistic (0,1) in the geographical detector was used to express the effect of each factor on hs-CRP level variation. It is calculated using the following mathematical formulation [35]:

q = 1 − *SSW / SST*



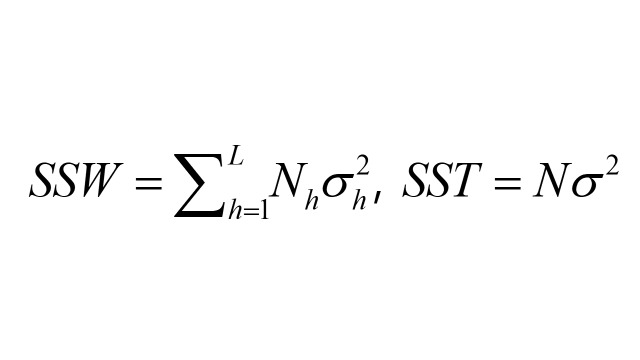



where h = 1, 2, …,; L is the number of layers of the selected risk factors X; N is the total number of samples; σ^2^ is the variance of Y (hs-CRP concentrations of middle-aged and older Chinese adults) in the whole study area; N_h_ is the mean number of samples of Y in layer h, and σ_h_^2^ is the local variance of Y in layer h. Therefore, SSW is the within sum of squares, and SST is the total sum of squares. The range of the q-statistic is 0–1; the larger the q, the stronger the effect of factor X on Y. As the q-statistics of different factors represent their explanatory power on Y separately, they are not additive.

To express the effect of single risk factors, the interactive effects of every pair of different factors were revealed by the interaction detector of the geographical detector. We can calculate the q-statistic of the interaction (q(X1∩X2)) of any pair of risk factors (X1, X2) and further compare q(X1∩X2) with q(X1) and q(X2) to evaluate whether their interaction can enhance or weaken the independent effect of each single risk factor on hs-CRP levels or whether those two factors are independent.

All statistical analyses were conducted using *R*, version 3.6.0 (R Foundation for Statistical Computing, Vienna, Austria). Bilateral *P*-values less than 0.05 were considered significant. The geographic detector method is implemented using the *R* software package.

## RESULTS

Demographic characteristics of participants are presented in Table S2 in the **Online Supplementary Document**. Of 10 308 participants, 51.86% were aged ≥ 60 years, and 48.14% were aged 45–60 years. The proportion of females was slightly higher than that of males. Overall, 5480 (53.16%) participants lived in the north and 4828 (46.84%) in the south. The mean concentration of hs-CRP of participants living in southern China were higher than that measured in those living in northern China (1.91 mg/L *vs*. 1.84 mg/L, *P* < 0.01). In southern China, males were more likely to have high hs-CRP levels. In the north, hs-CRP levels were higher in participants ≥ 60 years than in those aged 45–60 years (Table S3 in the **Online Supplementary Document**).

**Figure 2:**
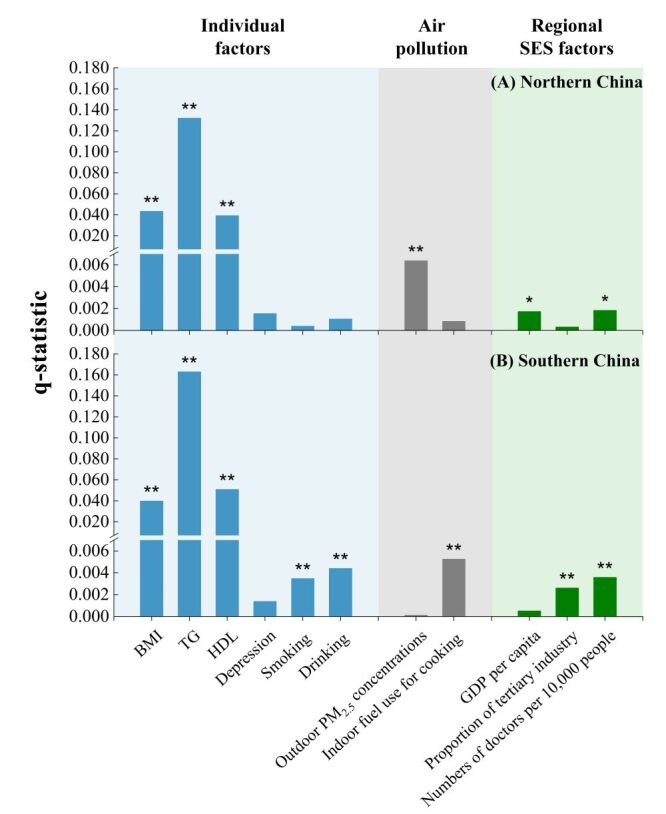
Explanatory power of various risk factors in hs-CRP levels by region (northern and southern China). BMI – body mass index, GDP – gross domestic product, HDL – high-density lipoprotein, hs-CRP – high-sensitivity C-reactive protein, PM_2.5 _– particulate matter, SES – socioeconomic status, TG – triglyceride.

Regarding the explanatory power of all the included risk factors (**Figure 2**), TG, BMI, and HDL levels were the top three leading factors in all populations. Of these, TG was the most significant and had a greater effect in the south than in the north, in males than in females, and in adults aged 45–60 years than in those aged ≥ 60 years.

High-sensitivity C-reactive protein levels were also significantly affected by other factors (**Figure 2**), and the results differed by region. In the north, outdoor PM_2.5_ concentrations, GDP *per capita*, and number of doctors per 10 000 individuals had a significant effect on hs-CRP levels. In the south, hs-CRP levels were significantly affected by risk factors, including indoor fuel used for cooking and drinking, number of doctors per 10 000 individuals, smoking, and proportion of tertiary industry, which are almost completely different from those in the north, except for the number of doctors per 10 000 individuals. Regional SES factors were significantly associated with hs-CRP levels in participants in the north and south, and their explanatory powers differed by region.

**Figure 3:**
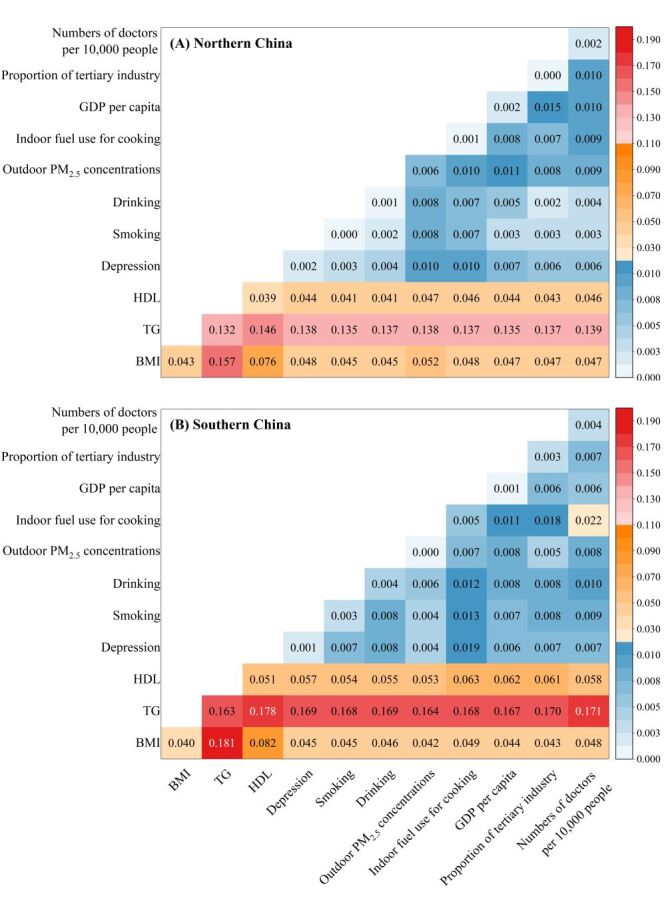
Interactive effects of paired risk factors on hs-CRP levels by region. **Panel A.** Northern China. **Panel B.** Southern China. BMI – body mass index, GDP – gross domestic product, HDL – high-density lipoprotein, hs-CRP – high-sensitivity C-reactive protein, PM_2.5 _– particulate matter, TG – triglyceride.

In the subgroup populations (Table S4 in the **Online Supplementary Document**), regional SES factors had a greater effect on participants aged 45–60 years than on those aged ≥ 60 years. Indoor fuel used for cooking had a greater effect on females than males and on older persons aged ≥ 60 years than those aged 45–60 years, especially in the south. The highest explanatory power of indoor air pollution on hs-CRP levels was found in females ≥ 60 years in the south (q = 0.015, *P* < 0.01), which was 2–3 times higher than that of other populations. The interactive effects of each paired risk factor on hs-CRP levels in different regions are shown in **Figure 3**, Panels A–B and in Figure S4–11 in the **Online Supplementary Document**, for each subgroup population in different regions. Overall, the explanatory power of any two independent risk factors was enhanced after the interaction. Triglyceride played a great role in the interaction effect. The dominant interactive power (*i.e*. the top 10 largest q-statistics) was TG interacting with 10 other factors without regional differences.

Figure S2 in the **Online Supplementary Document** illustrates the detailed trends of the top 10 dominant interaction effects for each subgroup population in different regions. For most populations, the strongest interaction effect was found between TG and BMI. Other leading interaction effects were found between TG and HDL, indoor air pollution, and depression. It is worth noting that although the independent explanatory power of depression was not significant for most subgroups in this manuscript, its interactive effect with TG was sometimes dominant (Figure S4–11 in the **Online Supplementary Document**). Moreover, regional SES factors played an important role in the interaction effects. Regional SES factors, such as proportion of tertiary industry, and the number of doctors per 10 000 individuals were observed to have a greater interaction effect with TG on hs-CRP levels than certain individual factors and air pollution.

## DISCUSSION

We assessed the role of individual factors, air pollution, and regional SES on hs-CRP levels, which is a powerful cardiovascular risk predictor, in different regions and identified their interactions.

Triglyceride was the most dominant for all subgroup populations without regional differences. This improves understanding of CVD [53,54]. Lipid levels are risk factors for cardiovascular events [53], with attention on low-density lipoprotein; low-density lipoprotein-lowering drugs are recommended for patients with heart disease [55]. However, recent research studies have challenged existing views and suggested that TG is an additional risk factor for CVD, and inflammation, (marked by hs-CRP levels) may play a connecting role [53]. In line with this, we observed a strong association between TG and hs-CRP levels, particularly in southern China suggesting that targeting TG levels in middle-aged and older Chinese adults should be prioritised to prevent CVD and promote health.

Particulate matter exposure is associated with an increased risk of CVD. Particulate matter– induced systemic inflammation, shown by enhanced hs-CRP level, is an important biological pathway [23,56–58]. Particulate matter exposure in the pulmonary bronchial tree may trigger a local inflammatory reaction with cytokine production, whose diffusion in the systemic circulation leads to an inflammation cascade. Particulate matter could also directly enter the vascular tree, interacts with endothelial cells and the immune system, and further activates the inflammation cascade [56,59,60]. Studies have explored the relationship between PM exposure and hs-CRP levels, but the results are inconsistent. In our study, the association between PM_2.5 _exposure and hs-CRP levels varies by region. Outdoor PM_2.5_ exposure significantly affected hs-CRP levels in northern China but not in the south. This is in line with a recent study that showed that people living in less economically developed counties are more vulnerable to ambient PM_2.5_ exposure than those in developed ones [18], considering that the regional GDP *per capita* in northern China is significantly lower than that in the south (46 450 *vs*. 49 790 Chinese Yuan, *P* < 0.01) (Table S2 in the **Online Supplementary Document**). This disparity may also be due to the higher PM_2.5_ concentrations in the north (60.23 *vs*. 44.99 micrograms per cubic meter (μg/m^3^), *P* < 0.01) (Table S2 in the **Online Supplementary Document**), and be associated with differences in PM composition as mentioned in previous studies [19].

Indoor air pollution also affected hs-CRP levels, and the results varied regionally. Most studies on the associations between air pollution and hs-CRP focus on outdoor air pollution, ignoring indoor air quality [15,17,21–24]. We observed that in the south, hs-CRP levels are more related to indoor cooking fuel use than outdoor PM exposure. We also observed that among people ≥ 60 years and females, hs-CRP levels were more linked to indoor cooking fuel use than among 45–60 years old and males. This finding is consistent with those of previous studies suggesting that indoor air quality is associated with older age, as they spend more time indoors after retirement, resulting in longer exposure to indoor air pollution [38]. Females logged more hours of cooking than men and were therefore exposed to a greater extent of indoor air pollution [45]. This may explain why the hs-CRP level was lower in females than in men in southern China. The relatively higher utilisation rate of clean fuel in southern China (Table S2 in the **Online Supplementary Document**) may contribute to reducing the hs-CRP level in females. The gender difference in hs-CRP remains controversial [61]. We observed that males had higher hs-CRP levels than females in southern China, which aligns with several previous studies [61,62]. The underlying mechanism of this sex disparity is still uncertain, but previous research indicates that lower BMI and lower smoking rates among females are related to decreased hs-CRP levels [61]. Our findings, emphasise the impact of indoor air pollution on hs-CRP in females, offering a new perspective on the sex difference in hs-CRP levels.

Our results stress that indoor air pollution, like outdoor air pollution, can significantly impact hs-CRP levels and should not be neglected. To effectively mitigate the effects of air pollution, both outdoor and indoor air pollution should attract the attention of policymakers, however, different priorities are needed for the north and south. In the north, the impact of outdoor air pollution is more serious. It is necessary to continue reducing the emission of outdoor air pollutants, increase blue and green space to improve air purification capacity, and improve residents' awareness of prevention to reduce outdoor activities in periods of heavy air pollution. In the south, more attention should be paid to indoor air pollution. Continuing to encourage the use of clean energy and improving energy efficiency, especially in underdeveloped areas, is necessary [63]. Promoting the use of natural gas in the countryside and building an energy supply model in which natural gas is used in conjunction with solar, wind and other bioenergy sources are the core of the policy.

We observed significant effects of all the selected regional SES factors on the hs-CRP levels of middle-aged and older Chinese adults, including GDP *per capita*, proportion of tertiary industry, and number of doctors per 10 000 individuals. Consistent with our findings, a study involving populations from Portugal and Switzerland, which have significant socioeconomic differences, indicated that SES was significantly associated with hs-CRP levels. Moreover, compared with other inflammatory biomarkers, this association was the most consistent in both cohorts [64]. Another global review pointed out socio-economic disparities in inflammatory multi system syndrome measured by CRP and other inflammatory markers [65]. However, due to the lack of comparable regional SES data, most of previous studies were based on individual SES, which is regarded as a result of the regional socioeconomic environment wherein individuals live and develop [66–70]. Within the context of China's diverse regional development, this is the largest national study that has examined the relationship between regional SES and hs-CRP levels and measured the influence of relevant factors quantitatively. Our findings can provide evidence of the relationship of residents’ health conditions to regional economic levels and healthcare resources, consistent with that from previous studies [28,46].

Notably, the most dominant socioeconomic factor of hs-CRP levels in middle-aged and older Chinese adults is the proportion of tertiary industry, which represents the regional industrial structure throughout China (Figure S3 in the **Online Supplementary Document**). This suggests that compared to the economic level, which is indicated by GDP *per capita*, the regional industrial structure has stronger links to hs-CRP levels. This is because, compared to macro-economic level, the regional industrial structure better reflects and plays a more important role in the improvement of quality and efficiency of economic growth, which benefits the residents’ health conditions based on lower energy consumption, lower environmental pollution, and better living conditions with the same economic level [29]. Geographically, the hs-CRP levels of middle-aged and older Chinese adults in southern China, where the regional GDP *per capita* was significantly higher than in northern China (49 790 *vs*. 46 450 Chinese Yuan, *P* < 0.01) (Table S2 in the **Online Supplementary Document**), were more strongly affected by the regional industrial structure. This implies the stronger effects of industrial structure on the hs-CRP levels of middle-aged and older Chinese adults living in regions with higher economic levels. Based on the impact of SES on hs-CRP, we suggest that policymakers should continue to increase investment in public health to improve the quality and coverage of healthcare. The policy of the more developed areas should focus on the quality and efficiency of economic growth, and the underdeveloped areas should still take economic development as the primary purpose.

Taking advantage of geographical detector, we further explored the interaction of different risk factors on hs-CRP levels in middle-aged and older Chinese adults [34]. The interaction between any two factors was enhanced. This indicates that when two risk factors are combined, they may have a stronger influence on hs-CRP levels than either factor alone. A strong interaction between regional SES factors (specifically, regional industrial structure and healthcare capacity) and TG was observed. Regional industrial structure can interact with TG because of its effect on air quality. Regional industrial structure is of crucial importance in reducing air pollution [18,29]. Air pollution is strongly associated with an increase in TG [71], which may further influence hs-CRP levels [53]. Regarding healthcare resources, adequate healthcare capacity (represented by the number of doctors per 10 000 people) can assist people in preventing and treating TG-related diseases and maintaining a normal TG level by providing sufficient and effective treatment and by equipping people with health knowledge to regulate TG levels [27]. Conversely, lack of health resources may lead to low awareness and treatment rates of elevated TG levels [72], and further have a combined effect on hs-CRP. The enhanced interaction between any two risk factors observed in our study implies that the hs-CRP level in middle-aged and older Chinese adults was affected by a combination of individual, environmental, and regional SES with regional and population differences.

This study has some limitations. First, we obtained only cross-sectional data; therefore, our findings were only statistically extrapolated. The underlying mechanisms of the effects of regional SES on hs-CRP levels in middle-aged and older Chinese adults may be complex and require longitudinal approaches for further study. Second, we explored only the regional variance between southern and northern China due to data limitations. Thus, the risk factors of the spatial variation of hs-CRP levels and the disparities of leading factors should be further studied on a finer scale. Third, we included only middle-aged and older Chinese adults. When extrapolating results to populations with different genetic characteristics, caution should be exercised. Fourth, the indoor air quality assessment was based on self-reporting. Therefore, errors caused by recall bias could exist. To reduce bias, we suggest that in the future CHARLS survey, investigators can directly identify the types of indoor cooking fuels and measure indoor air pollution concentrations with the help of instruments. Finally, other factors such as sleep duration, neighbourhood facilities, and other air pollutants (*e.g*. sulphur dioxide and ozone), that could also affect hs-CRP levels were not included because of missing relevant data; therefore, further studies are warranted.

## CONCLUSIONS

In conclusion, the leading risk factors for hs-CRP levels, a powerful cardiovascular risk predictor, in middle-aged and older Chinese adults varied regionally. Blood triglyceride, indoor air pollution and regional industrial structure had stronger effect on hs-CRP levels in the south, whereas smoking and drinking, outdoor air pollution and macro-economic level had greater effect in the north. Regional SES had significant effects on the hs-CRP levels of middle-aged and older Chinese adults through direct effects and interaction effects with other risk factors. The combined effect of individual and regional environment enhanced the explanatory power of each single risk factor. Policymakers should focus on the regional disparity of factors of cardiovascular risk predictor levels and to the combined effect of these factors, to devise strategies for effective regulation of cardiovascular risk predictor levels among middle-aged and older Chinese adults for CVD prevention.

## Additional material


Online Supplementary Document
