# Supplementary material for: The role of individual and regional environment factors on levels of a cardiovascular risk predictor in middle-aged and older Chinese adults
Source: J Glob Health. 2025 May 16;15:04131. doi: 10.7189/jogh.15.04131 (PMC12082255; doi:10.7189/jogh.15.04131)
Supplement: Online Supplementary Document [file jogh-15-04131-s001.pdf]

Supplement to: Li K, Wang P, Wang Z, Xu C, Wang S, Li Z, et al. The role of individual and regional environment factors on levels of a cardiovascular risk predictor in middle-aged and older Chinese adults. J Glob Health. 2025;15:04131.

Kexin Li[1]* (https://orcid.org/0000-0003-1529-821X), Peihan Wang[2,4]* (https://orcid.org/0009-0001-1359-9740), Zhenbo Wang[2,4]†(https://orcid.org/0000-0001-5342-0613), Chengdong Xu[3,4]†(https://orcid.org/0000-0002-9266-7309), Shaobin Wang[1] (https://orcid.org/0000-0002-0377-2095), Zhiyi Li[1] https://orcid.org/0009-0003-9646-2903), Peng Wang[1] (https://orcid.org/0009-0000-8699-6053)

[1]Key Laboratory of Land Surface Pattern and Simulation, Institute of Geographic Sciences and Natural Resources Research, Chinese Academy of Sciences, Beijing 100101, P.R. China;

[2]Key Laboratory of Regional Sustainable Development Modeling, Institute of Geographic Sciences and Natural Resources Research, Chinese Academy of Sciences, Beijing 100101, P.R. China;

[3]University of Chinese Academy of Sciences, Beijing, 100049, P.R. China;

[4]State Key Laboratory of Resources and Environmental Information System, Institute of Geographic Sciences and Natural Resources Research, Chinese Academy of Sciences, Beijing 100101, P.R. China

[*] Corresponding author:

Dr. Zhenbo Wang, E-mail: wangzb@igsnrr.ac.cn

at: Key Laboratory of Regional Sustainable Development Modeling, Institute of Geographic Sciences and Natural Resources Research, Chinese Academy of Sciences, Beijing 100101, P.R. China

Dr. Chengdong Xu, E-mail: xucd@lreis.ac.cn

at: State Key Laboratory of Resources and Environmental Information System, Institute of Geographic Sciences and Natural Resources Research, Chinese Academy of Sciences,

Beijing, P.R. China

Number of tables:	4
Number of Figures:	11

Table S1. North-south division of provinces in China

Region	Province
**North**	Beijing, Tianjin, Hebei, Henan, Shandong, Shanxi, Shaanxi, Heilongjiang, Jilin, Liaoning, Gansu, Ningxia Huizu Zizhiqu, Qinghai, Nei Mongol Zizhiqu, Anhui (Fuyang, Bozhou, Huaibei, Suzhou), Jiangsu (Xuzhou, Suqian, Lianyungang)
**South**	Anhui (except Fuyang, Bozhou, Huaibei, Suzhou), Jiangsu (except Xuzhou, Suqian, Lianyungang), Chongqing, Fujian, Guangdong, Guangxi Zhuangzu Zizhiqu, Guizhou, Hainan, Hubei, Hunan, Jiangxi, Sichuan, Yunnan, Zhejiang, Hong Kong SAR, Macau SAR, Taiwan
**Other**	Xizang Zizhiqu, Xinjiang Uygur Zizhiqu

Note: Anhui and Jiangsu are crossed by the Huai River, so the two provinces are also divided into northern and southern parts, respectively. Fuyang, Bozhou, Huaibei, Suzhou in Anhui province were divided into the north and the others into the south. Xuzhou, Suqian, Lianyungang in Jiangsu province were divided into the north and the others into the south.

Table S2. Statistical descriptions of demographic, socioeconomic and environmental variables in different regions of China

Variable		Subgroup	Overall N/Mean	Overall %/SD	North N/Mean	North %/SD	South N/Mean	South %/SD
N (Samples)			10308	100.00%	5480	53.16%	4828	46.84%
Age		≥60	5346	51.86%	2689	49.07%	2657	55.03%
		45-60	4962	48.14%	2791	50.93%	2171	44.97%
Gender		Male	4904	47.57%	2617	47.76%	2287	47.37%
		Female	5404	52.43%	2863	52.24%	2541	52.63%
hs-CRP (mg/L)			1.87	1.73	1.84	1.66	1.91	1.82
Individual characteristics	BMI		24.14	5.99	24.85	6.58	23.29	3.64
	Triglycerides (mg/L)		142.02	88.56	145.27	88.72	138.35	88.23
	High density lipoprotein (mg/dL)		51.15	11.36	49.56	10.66	52.95	11.86
	Depression (CESD-10 score)		9.07	5.21	9.14	5.23	8.97	5.19
	Smoking	Smoker	2903	28.16%	1577	28.78%	1326	27.46%
		Former smoker	1333	12.93%	759	13.85%	574	11.89%
		Nonsmoker	6072	58.91%	3144	57.37%	2928	60.65%
	Drinking	Drinker	3722	36.11%	1949	35.57%	1773	36.72%
		Former drinker	1091	10.58%	556	10.15%	535	11.08%
		Nondrinker	5495	53.31%	2975	54.29%	2520	52.20%
Air pollution	Indoor fuel use for cooking	Clean fuel	5853	56.78%	2713	49.51%	3140	65.04%
		Solid fuel	4355	42.25%	2727	49.76	1628	33.72%
		Other	100	0.97%	40	0.73%	60	1.24%
	Outdoor $PM_{2.5}$ concentrations (μg/m^3)		53.09	17.43	60.23	18.78	44.99	11.20
Regional SES factors	GDP per capita (Chinese yuan)		48018.37	25423.93	46455.67	22357.20	49792.11	28403.84
	Number of doctors per 10,000 people (person)		21.47	9.57	22.13	8.77	20.71	10.35
	Proportion of tertiary industry (%)		40.88	7.64	41.44	6.83	40.25	8.42

Note: Outdoor $PM_{2.5}$ concentrations, GDP per capita, number of doctors per 10,000 people, and proportion of tertiary industry were described based on annual city-level observations in 2015. SD: standard deviation.

Table S3. Statistical description of hs-CRP levels in participants, by sex, and ages

**Hs-CRP (mg/L)**		**Overall**		**North**		**South**	
		**Mean**	**p**	**Mean**	**p**	**Mean**	**p**
Sex	Male	1.90	0.09	1.81	0.316	2.00	<0.01
	Female	1.84		1.86		1.82	
Age	45-60	1.77	<0.01	1.76	<0.01	2.01	<0.01
	≥ 60	1.97		1.92		1.78	

Table S4. Explanatory power of risk factors on hs-CRP levels, by age and gender group in different regions of China

		**North**				**South**			
		**45-60**		**≥ 60**		**45-60**		**≥ 60**	
		**Male**	**Female**	**Male**	**Female**	**Male**	**Female**	**Male**	**Female**
Individual factors	BMI	0.049**	0.079**	0.027**	0,040**	0.060**	0.080**	0.021**	0.048**
	TG	0.213**	0.167**	0.100**	0.078**	0.243**	0.190**	0.145**	0.144**
	HDL	0.062**	0.047**	0.042**	0.035**	0.053**	0.053**	0.060**	0.046**
	Depression	0.006	0.001	0.006	0.008	0.007	0.005	0.007	0.002
	Smoking	0.000	0.001	0.001	0.005	0.004	0.001	0.004	0.002
	Drinking	0.005	0.003	0.004	0.005	0.007	0.008*	0.006*	0.002
Air pollution	Indoor fuel use for cooking	0.004	0.005	0.003	0.004	0.008	0.010	0.005	0.015*
	Outdoor $PM_{2.5}$ concentrations	0.009*	0.004	0.009*	0.008*	0.001	0.003	0.000	0.001
Regional socioeconomic factor	GDP per capita	0.010**	0.003	0.001	0.001	0.004	0.003	0.003	0.000
	Proportion of tertiary industry	0.004	0.001	0.002	0.001	0.010*	0.005	0.002	0.002
	Numbers of Doctors per 10,000 people	0.008*	0.002	0.001	0.005	0.005	0.015**	0.004	0.002

* q-statistic with significance p <0.05; **q-statistic with significance p <0.01

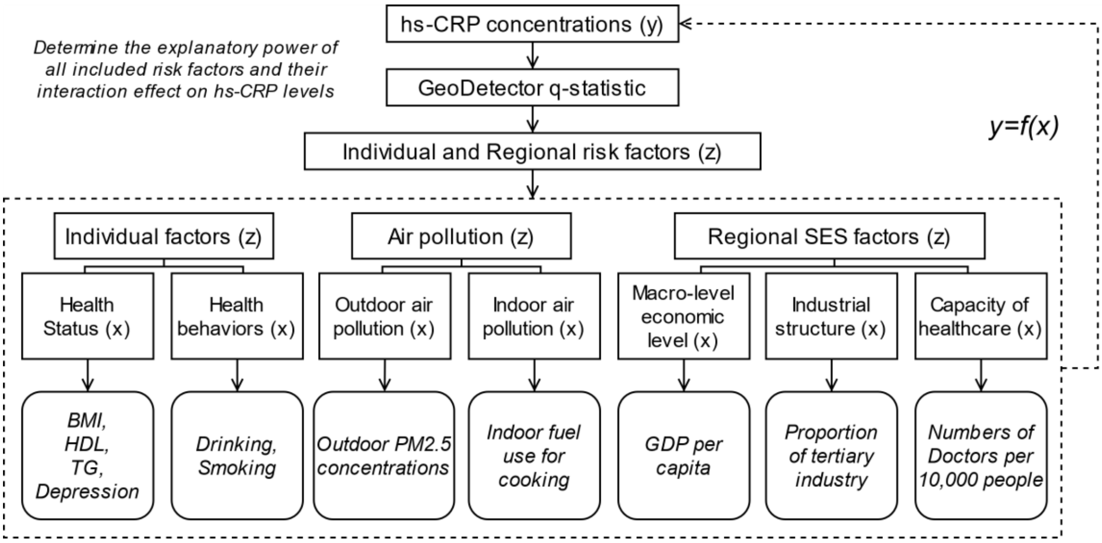

Figure S1. The relationship between hs-CRP concentrations and their proxy variables. BMI – body mass index, GDP – gross domestic product, HDL – high-density lipoprotein, hs-CRP – high-sensitivity C-reactive protein, PM2.5 – particulate matter, SES – socioeconomic status, TG – triglyceride.

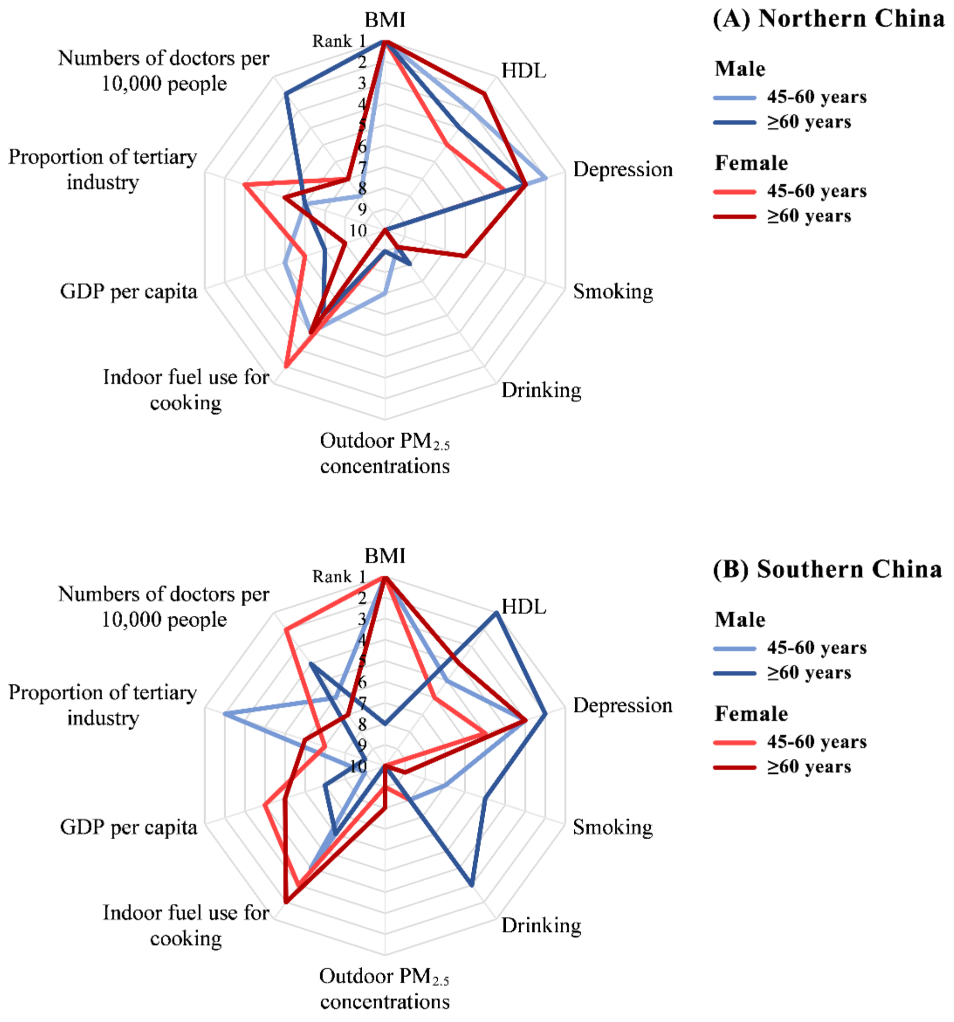

Figure S2. The top 10 q-statistics of each factor interacting with TG for the subgroup populations in different regions (Northern and Southern China) sorted by rank. BMI – body mass index, GDP – gross domestic product, HDL – high-density lipoprotein, PM2.5 – particulate matter, TG – triglyceride.

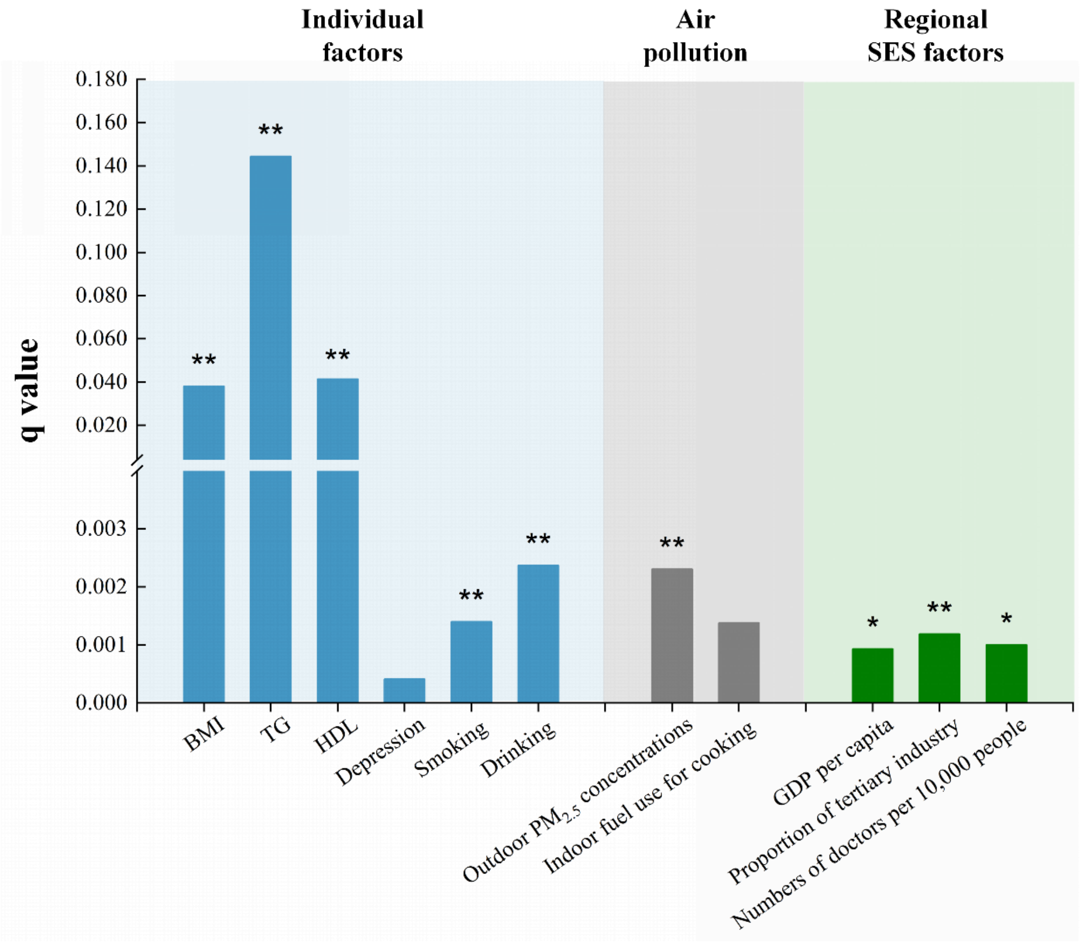

Figure S3. Explanatory power of various risk factors on hs-CRP levels in China

* q-statistic with significance p <0.05; **q-statistic with significance p <0.01

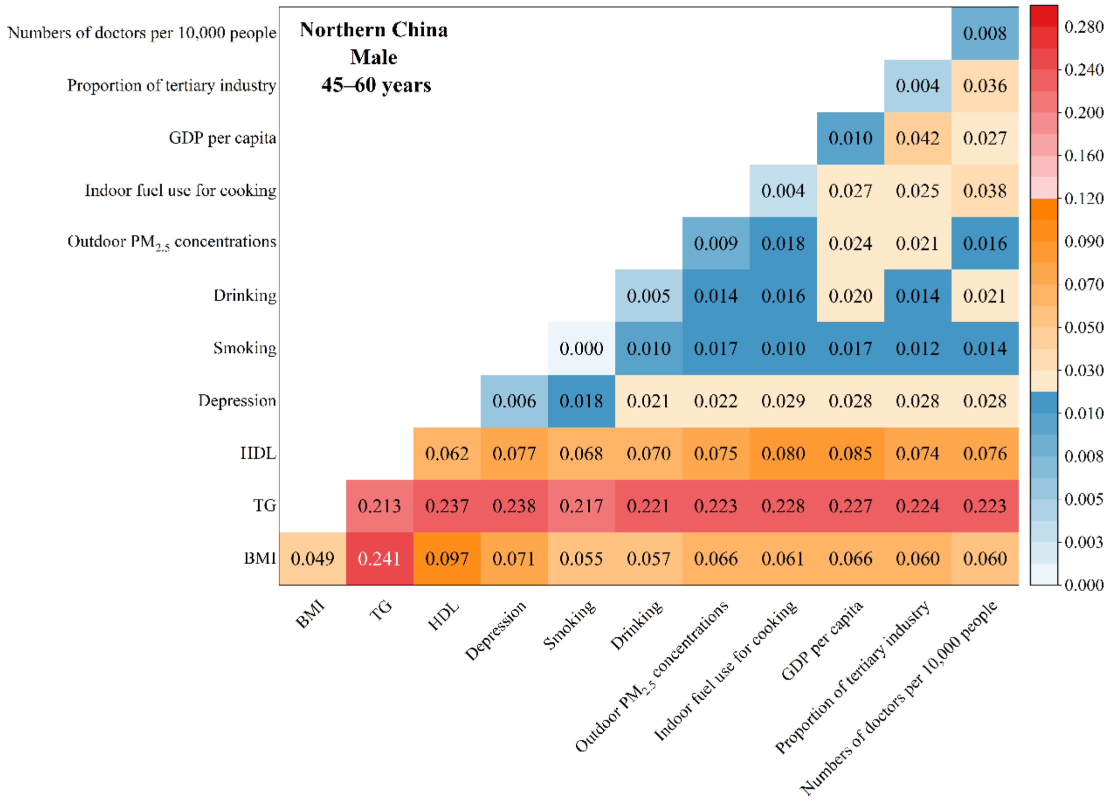

Figure S4. Interaction effects of each paired risk factors on hs-CRP levels of males aged between 45-60 years in northern China

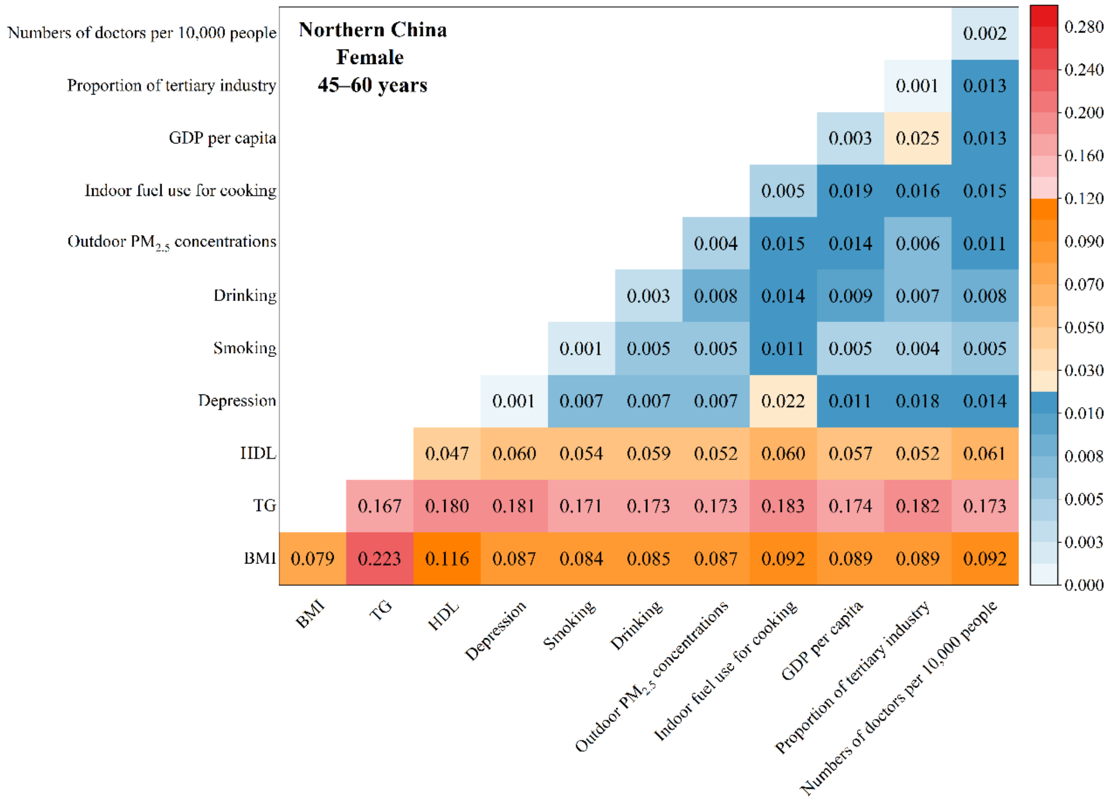

Figure S5. Interaction effects of each paired risk factors on hs-CRP levels of females between 45-60 years in northern China

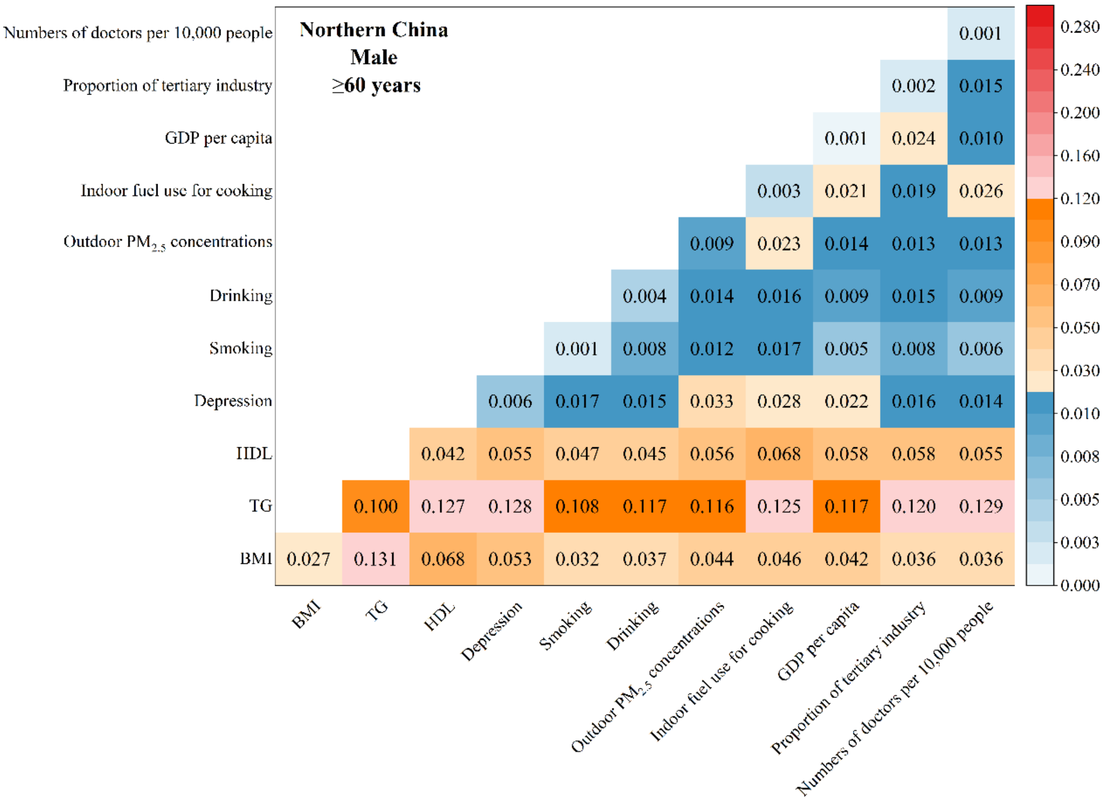

Figure S6. Interaction effects of each paired risk factors on hs-CRP levels of males aged ≥ 60 years in northern China

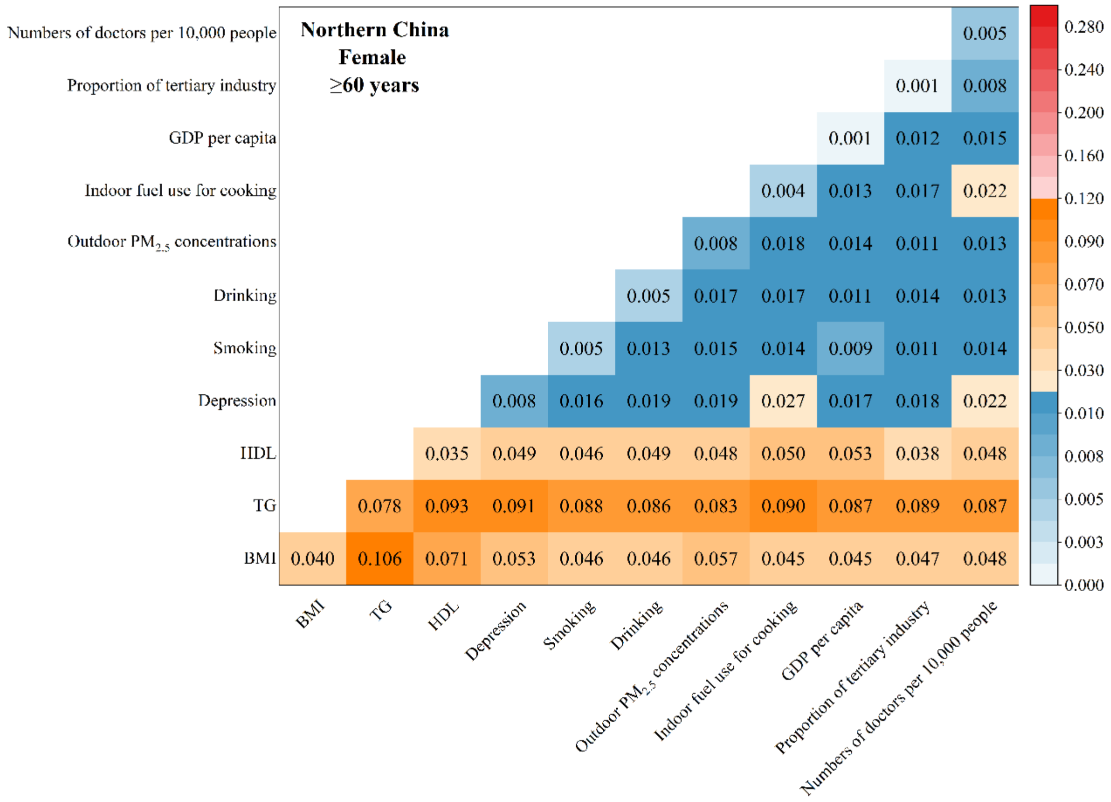

Figure S7. Interaction effects of each paired risk factors on hs-CRP levels of females aged ≥ 60 years in northern China

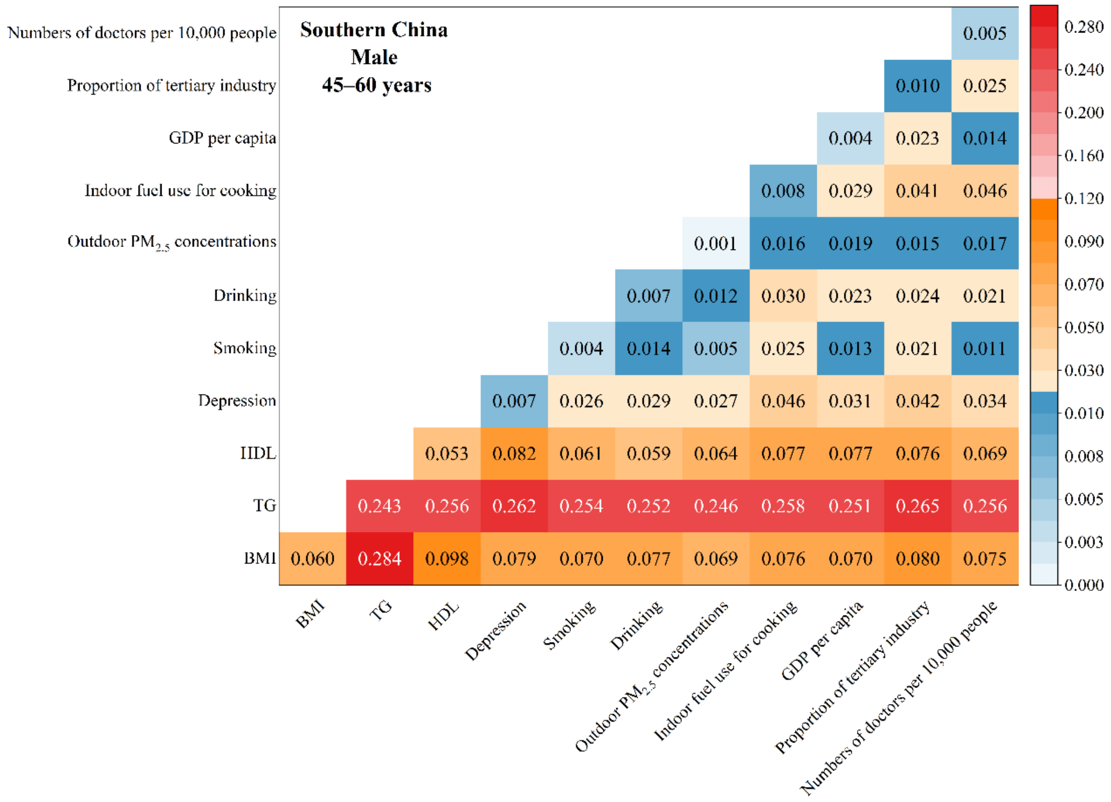

Figure S8. Interaction effects of each paired risk factors on hs-CRP levels of males between 45-60 years in southern China

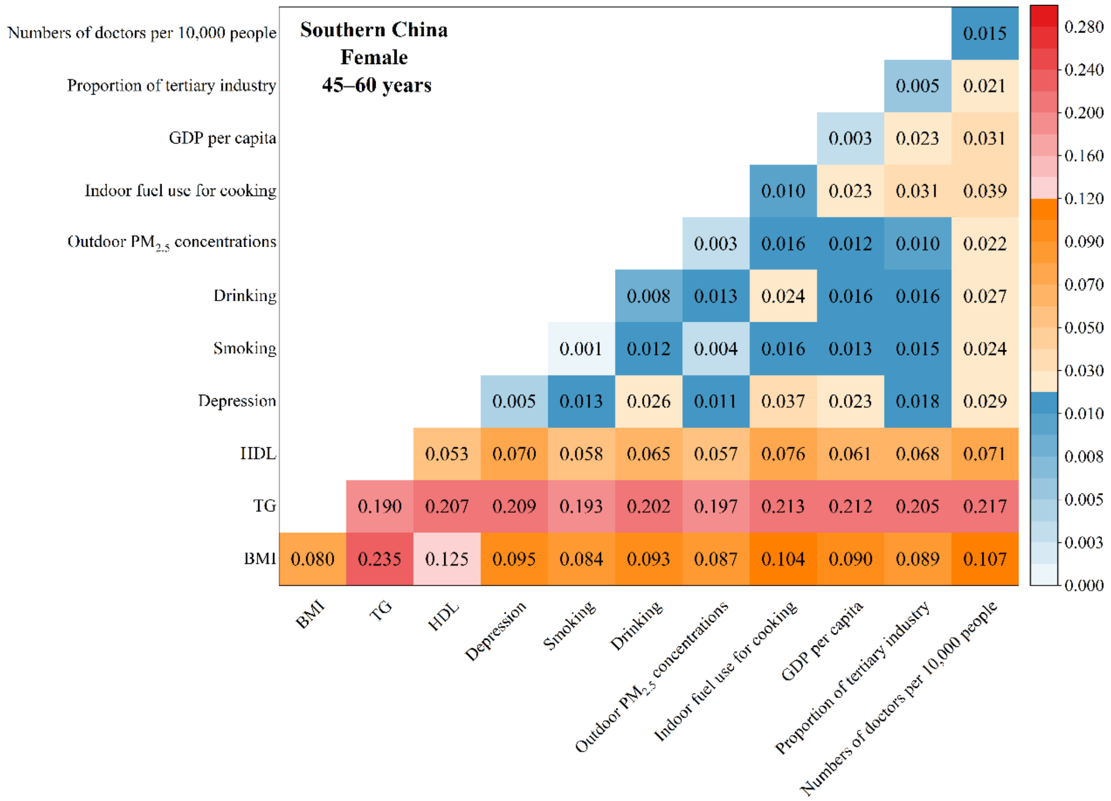

Figure S9. Interaction effects of each paired risk factors on hs-CRP levels of females aged between 45-60 years in southern China

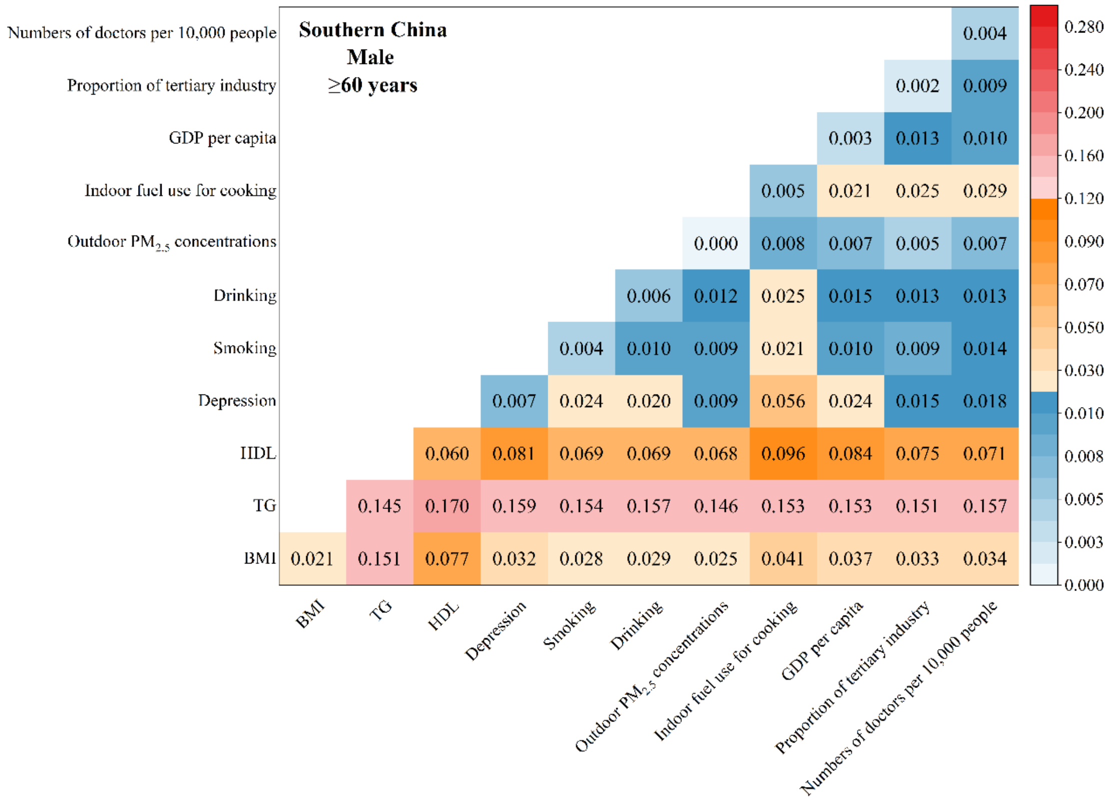

Figure S10. Interaction effects of each paired risk factors on hs-CRP levels of males aged ≥ 60 years in southern China

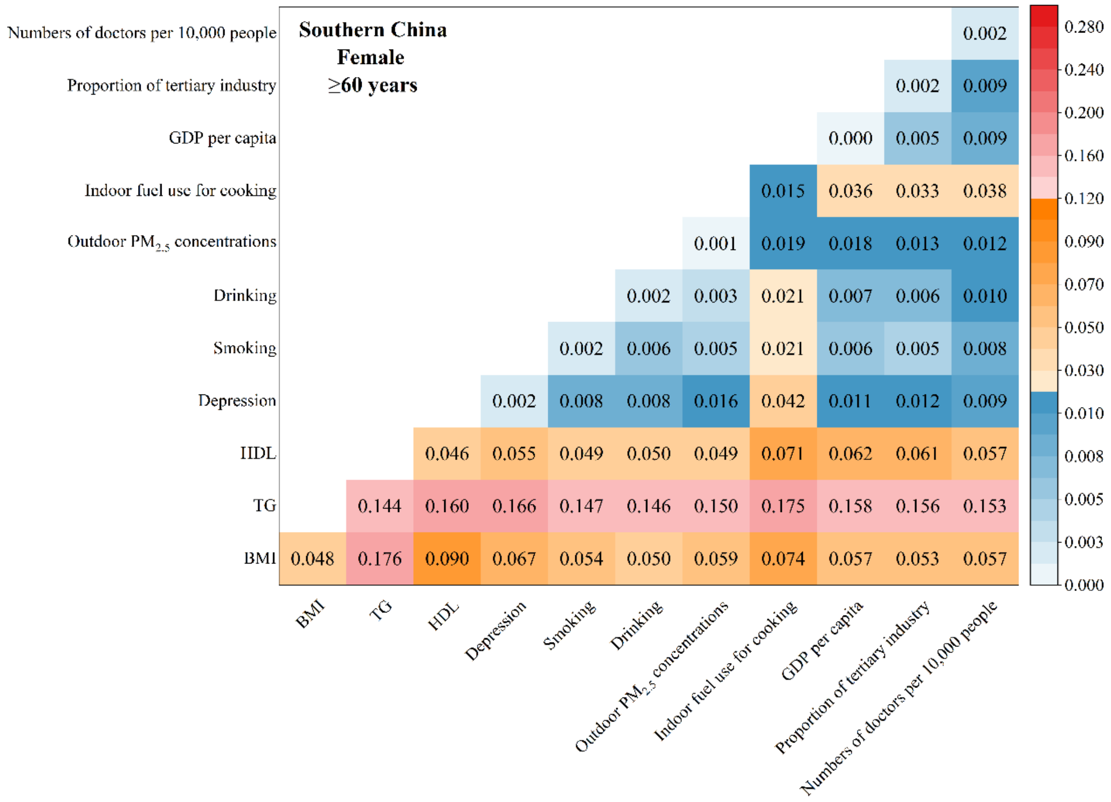

Figure S11. Interaction effects of each paired risk factors on hs-CRP levels of females aged ≥ 60 years in southern China
